# Phase 2, open-label extension (OLE) study of revusiran, an investigational RNAi therapeutic for the treatment of patients with transthyretin cardiac amyloidosis

**DOI:** 10.1186/1750-1172-10-S1-O21

**Published:** 2015-11-02

**Authors:** Julian D Gillmore, Rodney H Falk, Mathew S Maurer, Mazen Hanna, Verena Karsten, John Vest, Jared Gollob, Philip N Hawkins

**Affiliations:** 1 University College London Medical School, 1. National Amyloidosis Center, NW32PF, London, UK; 2 Brigham and Women's Hospital, 2. Amyloidosis Program, 02115, Boston, MA, USA; 3 NewYork-Presbyterian/Columbia, Clinical Cardiovascular Research Lab for the, 10034, New York, NY, USA; 4 Cleveland Clinic, 4. Heart and Vascular Institute, 44106, Cleveland, OH, USA; 5 5. Alnylam Pharmaceuticals, Clinical Development, 02142, Cambridge, MA, USA

## Background

In transthyretin (TTR) cardiac amyloidosis, myocardial deposition and accumulation of liver-derived TTR fibrils results in heart failure and death. The hereditary form of the disease is caused by mutations in the TTR gene and presents as familial amyloidotic cardiomyopathy (FAC), whereas senile systemic amyloidosis (SSA) is an acquired disease caused by wild-type TTR. There are currently no approved therapies available for ATTR cardiac amyloidosis. Revusiran is a liver-directed subcutaneously administered investigational RNA interference therapeutic comprised of a GalNAc-siRNA conjugate targeting both mutant and wild-type TTR mRNA. A Ph 2 study of revusiran in 26 patients with ATTR cardiac amyloidosis, in which revusiran was generally well tolerated and associated with > 85% sustained knockdown of serum TTR, was recently completed. A Ph 2 open label extension (OLE) of revusiran, available to all patients who participated in the Ph 2 trial, was initiated in November 2014.

## Methods

The primary objective of the OLE is to evaluate the long-term safety of 500 mg revusiran administered as 5 daily doses followed by weekly dosing for 2 years. Data on adverse events, laboratory assessments and ECG is collected. Secondary and tertiary objectives include serial assessments of pharmacodynamics, clinical outcomes including 6-Minute walk test, mortality, hospitalization, cardiac magnetic resonance imaging, 99mTechnetium scan, cardiac biomarkers and patient-reported QoL.

## Results

Patients who completed the Ph 2 trial include 12 patients with SSA and 14 patients with FAC (7 T60A, 5 V122I, 2 other). At the beginning of the Ph 2 study baseline data for these 26 patients included: mean age, 68 years; mean 6-minute walk distance, 408 meters; mean NT-proBNP, 3435 pg/mL; troponin I and T 0.13 and 0.045 ng/mL, respectively. The majority of patients had mild or moderate heart failure with NYHA class II (81%) and III (12%). All patients presented with intraventricular septal thickness (IVS) of > 15 mm (mean IVS 19 mm).

## Conclusions

As of June 8th 2015, 25 patients have been enrolled in the OLE. Interim 6-month data on safety, PD and clinical outcomes will be presented.

